# B- and T-Cell Responses After SARS-CoV-2 Vaccination in Patients With Multiple Sclerosis Receiving Disease Modifying Therapies: Immunological Patterns and Clinical Implications

**DOI:** 10.3389/fimmu.2021.796482

**Published:** 2022-01-17

**Authors:** Marco Iannetta, Doriana Landi, Gaia Cola, Laura Campogiani, Vincenzo Malagnino, Elisabetta Teti, Luigi Coppola, Andrea Di Lorenzo, Daniela Fraboni, Francesco Buccisano, Sandro Grelli, Marcello Mozzani, Maria Antonella Zingaropoli, Maria Rosa Ciardi, Roberto Nisini, Sergio Bernardini, Massimo Andreoni, Girolama Alessandra Marfia, Loredana Sarmati

**Affiliations:** ^1^ Infectious Disease Unit, Department of System Medicine, Tor Vergata University and Hospital, Rome, Italy; ^2^ Multiple Sclerosis Clinical and Research Unit, Department of Systems Medicine, Tor Vergata University and Hospital, Rome, Italy; ^3^ Department of Biomedicine and Prevention, Tor Vergata University and Hospital, Rome, Italy; ^4^ Department of Experimental Medicine, Tor Vergata University and Hospital, Rome, Italy; ^5^ Department of Public Health and Infectious Diseases, Sapienza University of Rome, Rome, Italy; ^6^ Department of Infectious Diseases, Istituto Superiore di Sanità (ISS), Roma, Italy; ^7^ Unit of Neurology, IRCCS Istituto Neurologico Mediterraneo NEUROMED, Pozzilli, Italy

**Keywords:** T-lymphocyte, peptides, IGRA, ocrelizumab, fingolimod, natalizumab, CD20, COVID

## Abstract

**Background:**

Vaccination campaign to contrast the spread of severe acute respiratory syndrome coronavirus-2 (SARS-CoV-2) has raised the issue of vaccine immunogenicity in special populations such as people with multiple sclerosis (PwMS) on highly effective disease modifying treatments (DMTs). While humoral responses to SARS-CoV-2 mRNA vaccines have been well characterized in the general population and in PwMS, very little is known about cell-mediated responses in conferring protection from SARS-CoV-2 infection and severe coronavirus disease-2019 (COVID-19).

**Methods:**

PwMS on ocrelizumab, fingolimod or natalizumab, vaccinated with two doses of mRNABNT162b2 (Comirnaty^®^) vaccine were enrolled. Anti-Spike (S) and anti-Nucleoprotein (N) antibody titers, IFN-gamma production upon S and N peptide libraries stimulation, peripheral blood lymphocyte absolute counts were assessed after at least 1 month and within 4 months from vaccine second dose administration. A group of age and sex matched healthy donors (HD) were included as reference group. Statistical analysis was performed using GraphPad Prism 8.2.1.

**Results:**

Thirty PwMS and 9 HDs were enrolled. All the patients were negative for anti-N antibody detection, nor reported previous symptoms of COVID-19. Peripheral blood lymphocyte counts were assessed in PwMS showing: (i) reduction of circulating B-lymphocytes in PwMS on ocrelizumab; (ii) reduction of peripheral blood B- and T-lymphocyte absolute counts in PwMS on fingolimod and (iii) normal B- and T-lymphocyte absolute counts with an increase in circulating CD16+CD56+ NK-cells in PwMS on natalizumab. Three patterns of immunological responses were identified in PwMS. In patients on ocrelizumab, anti-S antibody were lacking or reduced, while T-cell responses were normal. In patients on fingolimod both anti-S titers and T-cell mediated responses were impaired. In patients on natalizumab both anti-S titers and T-cell responses were present and comparable to those observed in HD.

**Conclusions:**

The evaluation of T-cell responses, anti-S titers and peripheral blood lymphocyte absolute count in PwMS on DMTs can help to better characterize the immunological response after SARS-CoV-2 vaccination. The evaluation of T-cell responses in longitudinal cohorts of PwMS will help to clarify their protective role in preventing SARS-CoV-2 infection and severe COVID-19. The correlation between DMT treatment and immunological responses to SARS-CoV-2 vaccines could help to better evaluate vaccination strategies in PwMS.

## Introduction

Since December 2019 the Severe Acute Respiratory Syndrome CoronaVirus-2 (SARS-CoV-2) has spread worldwide, causing the coronavirus disease-2019 (COVID-19) (1). In 2020, new SARS-CoV-2 vaccines with different designs have been developed and authorized for human use (2). Specifically, the mRNA vaccine mRNABNT162b2 (Comirnaty^®^) has been widely employed in the Italian population, including fragile and immunocompromised subjects (3).

Data on specific anti-Spike (S) antibody production and their protective role in vaccinated subjects are accumulating, while little is known on the T-cell response after vaccination, especially in immunosuppressed subjects (4).

Several studies have demonstrated an impairment of anti-SARS-CoV-2 antibody production in people with multiple sclerosis (PwMS) receiving specific disease modifying therapies (DMTs), such as the sphingosine-1-phosphate receptor modulator fingolimod or the anti-CD20 drug ocrelizumab, after either natural infection or vaccination (5–7). Moreover, Achiron et al. and Sormani et al. showed poor seroconversion rates after SARS-CoV-2 vaccination in PwMS receiving ocrelizumab and fingolimod (8, 9), stimulating a debate on timing and recommendations on SARS-CoV-2 vaccination in PwMS on different DMTs (10, 11). T-cell specific immunity represents a fundamental aspect for the evaluation of vaccine response and protection from SARS-CoV-2. Recently, in a rhesus macaques model, McMahan et al. demonstrated that the depletion of CD8+ T-cells in convalescent animals, partially abrogated the protective efficacy of natural immunity in rechallenge with SARS-CoV-2, suggesting a role for cellular immunity in subjects with waning or sub-protective antibody titers (12).

We have previously investigated SARS-CoV-2 specific T-cell responses in PwMS on ocrelizumab treatment, healed from COVID-19, using an interferon (IFN)-γ release assay (IGRA) after overnight stimulation of whole blood with SARS-CoV-2 specific peptide libraries. Despite the lack of an antibody response to the natural infection, we were able to identify a specific T-cell response in all the healed subjects included in the study (13).

Here we aimed to characterize specific B- and T-cell responses towards the Spike protein of SARS-CoV-2 in PwMS receiving three different types of DMTs (ocrelizumab, fingolimod and natalizumab), after two-dose vaccination with the mRNA vaccine Comirnaty^®^, evaluating also the peculiar changes induced by the DMTs on peripheral blood lymphocyte counts.

## Methods

### Study Population and Blood Sampling

The study was approved by the local Ethic Committees (Protocol VaRIA-BT-SARS number 125.21) and conducted in accordance with the declaration of Helsinki. PwMS treated with ocrelizumab, fingolimod or natalizumab that received 2 doses of the mRNABNT162b2 (Comirnaty^®^) vaccine were enrolled in the study. Blood samples for anti-SARS-CoV-2 antibody assessment and T-cell stimulation were obtained on the same day, after at least one month and within 4 months from administration of the second dose of the vaccine. Prior history of symptomatic SARS-CoV-2 infection was considered an exclusion criterion. A group of 9 healthy donors (HD) vaccinated with two doses of Comirnaty^®^ was also included in the study. All subjects enrolled in the study signed a written informed consent.

### Anti-SARS-CoV-2 Serology

Specific SARS-CoV-2 total anti-Spike (S) and anti-Nucleocapsid (N) antibodies were measured with two commercial electrochemiluminescence immunoassays (ECLIA) (Elecsys^®^ Anti-SARS-CoV-2 S and Elecsys^®^ Anti-SARS-CoV-2, Roche Diagnostics) according to the manufacturer’s instructions. These tests detect predominantly IgG, but also IgA and IgM. The test for anti-S antibody assay has a linear range from 0,4 to 250 U/mL and a result ≥ 0,8 U/mL is considered positive. For the anti-N antibody ECLIA, a cutoff index (COI) ≥ 1,0 is considered reactive.

### T-, B-, NK- Lymphocyte Assessment in Peripheral Blood

Peripheral blood lymphocyte subsets were assessed with a lyse no wash standardized protocol, using the BD Multitest 6-Color TBNK Reagent and trucount tubes (BD Biosciences), as previously described (14).

### T-Cell Stimulation With SARS-CoV-2–Specific Peptide Libraries

T-lymphocyte specific response was assessed with an interferon (IFN)-γ release assay (IGRA) after overnight stimulation with SARS-CoV-2 peptide libraries. Pools of lyophilized peptides, consisting mainly of 15-mer sequences with 11 amino acids overlap, covering the immunodominant sequence domains of the Spike glycoprotein (S) (GenBank MN908947.3, Protein QHD43416.1) and the Nucleocapsid phosphoprotein (N) (GenBank MN908947.3, Protein QHD43423.2) of SARS-CoV-2 were purchased from Milteny Biotec. Specifically, PepTivator SARS-CoV-2 Prot_S1 covered the N-terminal S1 domain of the spike protein (amino acids [aa] 1–692). PepTivator SARS-CoV-2 Prot_S covered selected immunodominant sequence domains of the spike protein (aa 304–338, 421–475, 492–519, 683–707, 741–770, 785–802, and 885–1273). PepTivator SARS-CoV-2 Prot_N covered the complete sequence of the N phosphoprotein of SARS-CoV-2. Briefly, 500µl of fresh heparinized blood were incubated overnight at 37°C and 5% CO_2_, after stimulation with PepTivator S1, S, and N (3 conditions for each peptide library plus 1 condition with pooled peptides) at a final concentration of 1µg/ml. For each patient a negative and positive (phytohemagglutinin (PHA) 5µg/ml) control was also included. After 18 hours, supernatants were collected and stored at -80°C. IFN-γ production was assessed with a commercial enzyme linked immunosorbent assay (ELISA) kit (Human IFN-γ DuoSet ELISA, R&D Systems), following manufacturer’s instructions. IFN-γ response was defined as peptide or PHA stimulated condition minus unstimulated condition.

### Statistical Analyses

Data are represented as median with interquartile range (IQR). Differences among groups were assessed using the two tailed χ^2^ test for qualitative variables; for quantitative variables the non-parametric Kruskal-Wallis test with the Dunn’s multiple comparison post-test were used, reporting p values adjusted for multiple comparisons. Correlations between quantitative data were assessed using the non-parametric Spearman test. Results were considered statistically significant if p<0,05. Statistical analysis was performed using GraphPad Prism 8.2.1 for macOS.

## Results

### Study Population

A total of 30 patients (21 women and 9 men) with multiple sclerosis (PwMS) were enrolled in the study, with a median age of 41 years (IQR 33-46) (**Table 1**). All patients had completed a two-dose vaccination cycle with the Comirnaty^®^ vaccine within 4 months before blood sampling, and none tested positive for SARS-CoV-2 nor had ever reported COVID-19 symptoms before enrollment. The median number of days from the second dose of Comirnaty^®^ vaccine to blood sampling for anti-S antibody and specific T-cell response was 73 days (range 34–106 days). The relapsing remitting (RR) form of MS was the most represented in the cohort of PwMS (29/30) with only one primary progressive (PP). Median expanded disability status scale (EDSS) was 2 (IQR: 0-3) and median disease duration was 13 years [IQR: 7-15]. Concerning previous MS treatments, 5 patients were naïve for prior therapeutic regimens.

**Table 1:** Demographic and clinical characteristics of PwMS and healthy donors.

	HDN=9	Overall PwMSN=30	OCRN=10	FYTN=10	NATN=10	p*
Age median [IQR]	30 [30-31]	41 [33-46]	33 [30-47]	45 [41-48]	36 [32-43]	0.08
Sex M/F	4/5	9/21	5/5	3/7	1/9	0.15
MS type (RR/PP)	NA	29/1	9/1	10/0	10/0	0.36
EDSS median [IQR]	NA	2.0 [0-3.0]	2.0 [2.0-4.5]	1.5 [1.0-3.0]	0.5 [0.0-2.5]	0.29
Disease duration median years [IQR]	NA	13 [7-15]	9 [4-16]	15 [12-17]	10 [7-14]	0.17
Previous treatment for MS (Yes/No)	NA	25/5	8/2	9/1	8/2	0.79
Duration of current DMT median years [IQR]	NA		3 [2-5]	6 [5-7]	5 [1-7]	0.03
Days from DMT last administration to sampling median days [IQR]	NA		217 [197-232]	NA	42 [35-35-42]	NA

*Comparison of OCR, FYT and NAT groups with a 2-tailed χ^2^ test for qualitative data or the non-parametric Kruskal-Wallis test for quantitative data, as appropriate.

PwMS, people with multiple sclerosis; HD, healthy donors; OCR, ocrelizumab group; FYT, fingolimod group; NAT, natalizumab group; IQR, interquartile range; MS, multiple sclerosis; RR, relapsing remitting; PP, primary progressive; EDSS, expanded disability status scale; NA, not applicable; DMT, disease modifying treatment.

PwMS were stratified into three groups, according to the ongoing MS treatment: ocrelizumab (OCR), fingolimod (FTY) and natalizumab (NAT). None of the patients was on interferon. No serious adverse reactions were recorded after the vaccination. Median duration of current DMT was 3 [IQR: 2-5], 6 [IQR: 5-7] and 5 [IQR: 1-7] years for OCR, FYT and NAT respectively, with a statistically significant difference (p=0,03). When comparing the three groups, no statistically significant differences were found in age, sex distribution, EDSS, disease duration, rate of PwMS naïve for prior MS treatments (**Table 1**).

### SARS-CoV-2 Anti-N and Anti-S Antibodies

**Figure 1:**
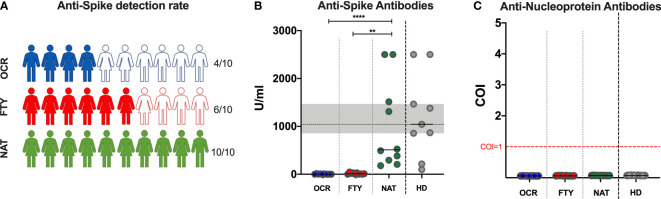
Detection of SARS-CoV-2 anti-Spike and anti-Nucleoprotein in PwMS and healthy donors. Specific SARS-CoV-2 anti-Spike detection rate **(A)** and titers **(B)**, and anti-Nucleoprotein **(C)** antibody detection in PwMS receiving ocrelizumab (OCR), fingolimod (FTY) and natalizumab (NAT). **(A)**. Color filled icons represent PwMS with anti-Spike titers above 0,8 U/ml, which was considered the positivity threshold. The differences among the three groups were assessed using the Kruskal-Wallis test, and the Dunn’s multiple comparison post-test, after correction for multiple comparisons. The level of statistical significance is reported. **(B)**. Horizontal continuous segments represent medians. Horizontal dotted black line and the gray area represent the median value and the interquartile range [IQR] observed in 9 healthy donors age and sex matched with PwMS. **(C)**. Horizontal dotted red line represents the cut-off value index (COI)=1, to discriminate positive from negative results. All the enrolled PwMS were negative for the detection of anti-Nucleoprotein antibodies. PwMS, people with multiple sclerosis; OCR, ocrelizumab group (blue); FYT, fingolimod group (red); NAT, natalizumab group (green); HD, healthy donors (gray). ** 0,01<p< 0,001; **** p<0,0001.

Anti-S and anti-N antibodies were assessed in all the enrolled subjects. In PwMS, anti-S antibody titers were either undetectable (<0,4 U/ml) or detectable below the lower cutoff of positivity (0,8 U/ml) in 6/10, 4/10 and 0/10 PwMS of the OCR, FTY and NAT group respectively, with a statistically significant higher rate of negative subjects in the OCR and FTY groups (χ^2^ p=0,02) (**Figure 1A**). Moreover, reduced anti-S titers were found in OCR and FTY groups compared to NAT group (Kruskal-Wallis p<0,0001. Post-test analysis, OCR vs FTY p=ns and FTY vs NAT p=0,0015 and OCR vs NAT p<0,0001). Median values observed in the NAT group were reduced compared to the values observed in healthy donors, although the difference was not statistically significant (**Figure 1B**). Overall, anti-S antibody titers were reduced in PwMS receiving ocrelizumab and fingolimod, compared to patients on natalizumab treatment, who had anti-S antibody production comparable to HD.

For anti-N antibodies, all the patients had non-reactive results at the ECLIA, with a COI<1 for all the tested samples (median 0,063; IQR: 0,059-0,070; range: 0,055-0,080) (**Figure 1C**).

### T-, B-, NK- Lymphocyte Assessment in Peripheral Blood

T-, B- and NK-cell absolute counts were assessed in all PwMS with a standardized protocol routinely used in clinical practice (14). Blood samples were collected simultaneously with samples tested for anti-SARS-CoV-2 serology and T-cell responses. Results were compared amongst different DMTs group, with healthy donors and with laboratory reference values.

**Figure 2:**
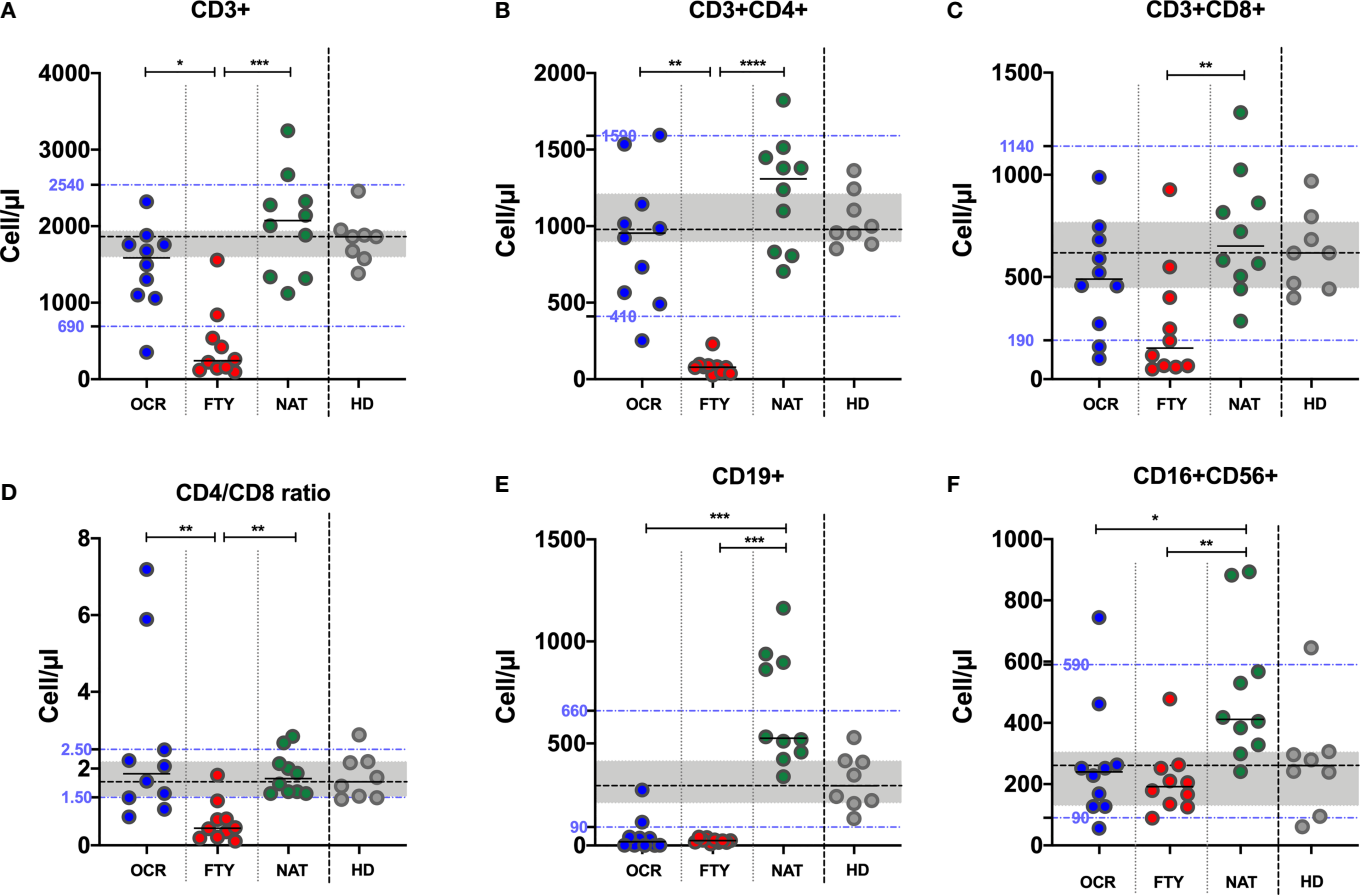
Peripheral blood lymphocyte absolute counts and CD4/CD8 ratio in PwMS on DMTs and HD. Peripheral blood CD3+ **(A)**, CD4+ **(B)**, CD8+ **(C)**, CD19+ **(E)**, CD16+CD56+ **(F)** lymphocyte absolute counts and CD4/CD8 ratio **(D)** were assessed in PwMS. The differences among the three groups were assessed using the Kruskal-Wallis test, and the Dunn’s multiple comparison post-test, after correction for multiple comparisons. The level of statistical significance is reported. **(A–E)**. Horizontal continuous segments represent medians. Horizontal dotted black line and the gray area represent the median value and the interquartile range [IQR] observed in 9 healthy donors age and sex matched with PwMS. Horizontal dotted blue lines represent the lower and upper limit of the normality range of the laboratory for peripheral blood lymphocyte absolute counts. PwMS, people with multiple sclerosis; OCR, ocrelizumab group (blue); FYT, fingolimod group (red); NAT, natalizumab group (green); HD, healthy donors (gray). * p<0,05; ** 0,01<p< 0,001; *** 0,001<p< 0,0001; **** p<0,0001.

Concerning T-lymphocytes, total CD3+ cell absolute counts were reduced in the FYT group compared to OCR and NAT groups (Kruskal-Wallis p=0,0002. Post-test analysis, OCR vs FTY p=0,0248 and FTY vs NAT p=0,0001 and OCR vs NAT p=ns). Total CD3+ cell absolute counts of PwMS in the FTY group were also reduced compared to HD (p<0,0001) and below the lower limit of the laboratory normality range in 8 out of 10 subjects (**Figure 2A**). Similarly, CD3+CD4+ cell absolute counts were reduced in the FYT group compared to OCR and NAT groups (Kruskal-Wallis p<0,0001. Post-test analysis, OCR vs FTY p=0,0024 and FTY vs NAT p<0,0001 and OCR vs NAT p=ns). CD3+CD4+ cell absolute counts of PwMS in the FTY group were also reduced compared to HD (p<0,0001) and below the lower limit of the laboratory normality range in all the FTY subjects (**Figure 2B**). CD3+CD8+ cell absolute counts were reduced in the FTY group compared to NAT group (Kruskal-Wallis p=0,0077. Post-test analysis, OCR vs FTY p=ns and FTY vs NAT p=0,0058 and OCR vs NAT p=ns). CD3+CD8+ cell absolute counts of PwMS in the FTY group were also reduced compared to the values observed in HD (p=0,009) and below the lower limit of the laboratory normality range in 6 out of 10 FTY subjects (**Figure 2C**). CD4/CD8 ratio values were reduced in PwMS of the FTY group, compared to OCR and NAT group (Kruskal-Wallis p=0,0005. Post-test analysis, OCR vs FTY p=0,0029 and FTY vs NAT p=0,0020 and OCR vs NAT p=ns). CD4/CD8 ratio values were also reduced in the FYT group compared to HD (p=0,0009) and below the lower limit of the laboratory normality range (**Figure 2D**).

Concerning B-lymphocytes, CD19+ cell absolute counts were reduced in the OCR and FTY groups compared to NAT group (Kruskal-Wallis p<0,0001. Post-test analysis, OCR vs FTY p=ns and FTY vs NAT p=0,0003 and OCR vs NAT p=0,0007). Furthermore, CD19+ lymphocyte absolute counts were reduced in the OCR and FTY groups and increased in the NAT group compared to HD (p=0,0005; p<0,0001 and p=0,0031 respectively). CD19+ lymphocyte absolute counts were also below the lower limit of the laboratory normality range in the OCR and FTY groups (8/10 and 10/10, respectively) (**Figure 2E**).

Concerning NK lymphocytes, CD3-CD16+CD56+ cell absolute counts were reduced in OCR and FTY groups compared to NAT group (Kruskal-Wallis p=0,0036. Post-test analysis, OCR vs FTY p=ns and FTY vs NAT p=0,0047 and OCR vs NAT p<0,0319). NK-cell absolute counts were comparable to HD values in OCR and FTY groups and increased in the NAT group, compared to HD (p=0,68, p=0,32 and p=0,016, respectively). Median values of NK-cell absolute counts were within the reference values of the laboratory for all the three groups of PwMS (**Figure 2F**).

Overall, in PwMS receiving ocrelizumab therapy, there was a marked reduction of peripheral blood B-lymphocyte absolute counts, while in patients on fingolimod treatment both peripheral B- and T-lymphocyte absolute counts were reduced. Although peripheral blood total CD3+, CD4+ and CD8+ T-lymphocyte absolute counts were increased in PwMS on natalizumab compared to HD, the difference did not reach the statistical significance (probably due to the limited number of patients). Conversely, CD19+ B-cell and CD16+CD56+ NK cell absolute counts were significantly increased in PwMS on natalizumab compared to HD.

### T-Cell Stimulation With SARS-CoV-2–Specific Peptide Libraries

After overnight stimulation of whole blood samples with SARS-CoV-2 S1, S and N peptide libraries, interferon gamma production in supernatants was detected with a commercial ELISA kit and the results were analyzed comparing OCR, FYT and NAT groups. Results obtained from healthy donors were used as a reference.

**Figure 3:**
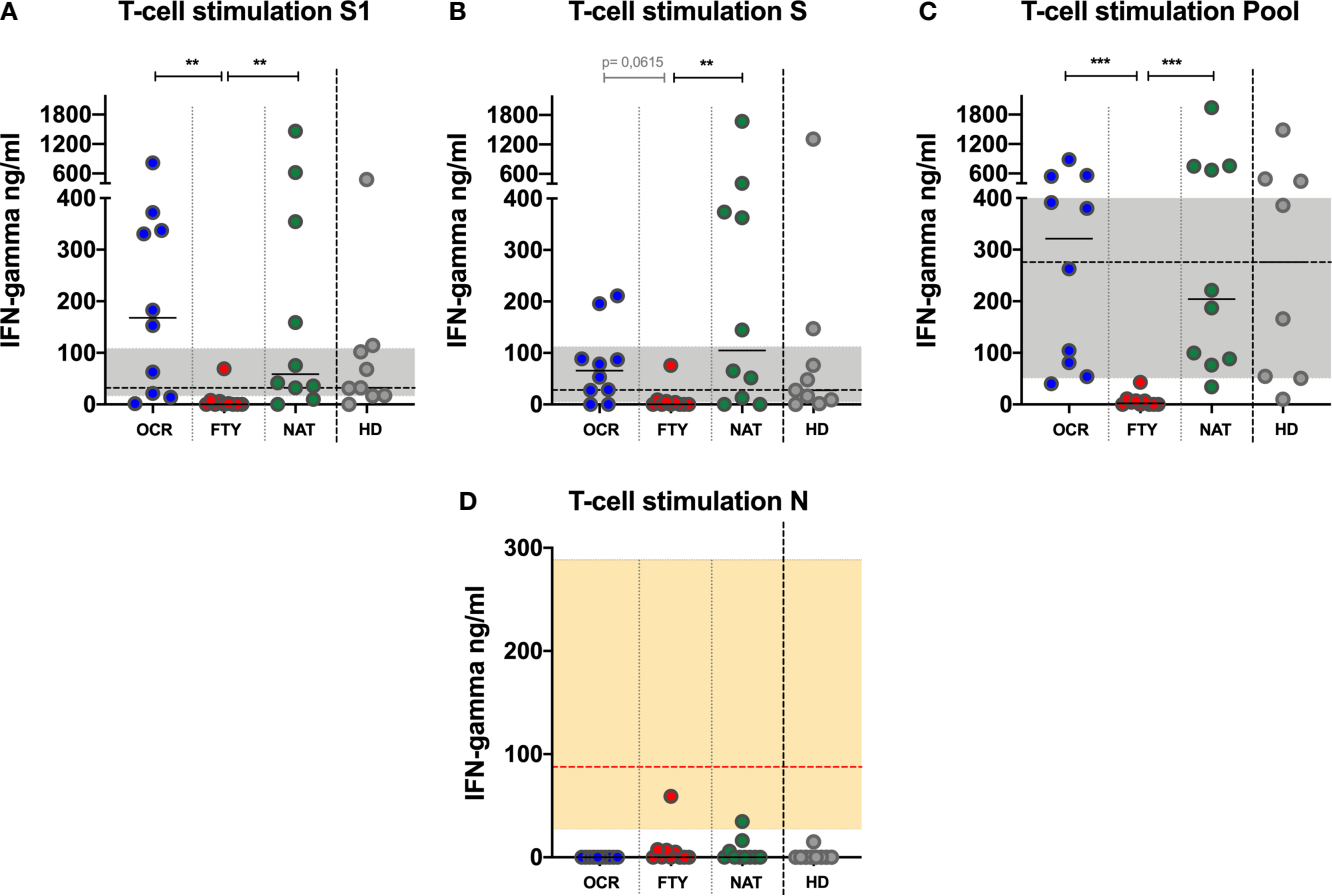
IFN-γ production upon Spike and Nucleocapside peptide libraries stimulation of whole blood of PwMS. IFN-γ production upon S1 **(A)**, S **(B)** and N **(D)** peptide libraries is represented for all the PwMS enrolled in the study. Furthermore, IFN-γ production upon pooled peptide (Pool=S1+S+N) is represented **(C)**. The differences among the three groups were assessed using the Kruskal-Wallis test, and the Dunn’s multiple comparison post-test, after correction for multiple comparisons. The level of statistical significance is reported. **(A–C)**. Horizontal continuous segments represent medians. Horizontal dotted black line and the gray area represent the median value and the interquartile range [IQR] observed in 9 healthy donors age and sex matched with PwMS. **(D)**. Horizontal dotted red line and the orange area represent the median (60,6 ng/ml) and IQR (16,3-346,3 ng/ml) observed in 5 PwMS on ocrelizumab treatment with symptomatic COVID-19 disease, which have been previously described (12). All the PwMS with the exception of 2 subjects (1 on fingolimod and 1 on natalizumab) showed IFN-γ production upon N stimulation comparable to those observed in the 5 PwMS on ocrelizumab with symptomatic COVID-19 disease. PwMS, people with multiple sclerosis; OCR, ocrelizumab group (blue); FYT, fingolimod group (red); NAT, natalizumab group (green); HD, healthy donors (gray). ** 0,01<p< 0,001; *** 0,001<p< 0,0001.

After S1 peptide library stimulation, a reduced IFN-γ production was detected in FYT group, compared to OCR and NAT (Kruskal-Wallis p=0,0013. Post-test analysis, OCR vs FTY p=0,0029 and FTY vs NAT p=0,0084 and OCR vs NAT p=ns) (**Figure 3A**). Similarly, upon S peptide library stimulation, reduced amounts of IFN-γ were detected in FYT group, compared to OCR and NAT (Kruskal-Wallis p=0,0069. Post-test analysis, OCR vs FTY p=0,0615 and FTY vs NAT p=0,0078 and OCR vs NAT p=ns) (**Figure 3B**). These results were confirmed after normalization of SARS-CoV-2 Spike peptide-induced to PHA-induced IFN-γ production (**Supplementary Data 1.1**), as proposed for IGRA assays for the diagnosis of active and latent tuberculosis (15).

After N peptide library stimulation, IFN-γ was undetectable in the majority of patients in the three DMTs groups (10/10 OCR, 6/10 FTY, 7/10 NAT). In 0/10 OCR, 4/10 FTY and 3/10 NAT subjects, a detectable IFN-γ production was observed, but lower than the IFN-γ production previously detected in 5 PwMS on ocrelizumab with symptomatic COVID-19, analyzed elsewhere by our group (12) (**Figure 3D**). This could be due to cross reactivity with other coronaviruses previously encountered by the patients, or asymptomatic SARS-CoV-2 prior infection. Aiming to exclude this second hypothesis, all the enrolled PwMS were tested for the detection of anti-SARS-CoV-2 N antibodies, which were undetectable in all the analyzed samples (**Figure 1C**). The undetectability of anti-N antibody in PwMS on ocrelizumab and fingolimod cannot definitively rule out a previous asymptomatic SARS-CoV-2 infection, due to the impairment of specific antibody production in these subjects.

To increase the sensitivity of the IGRA test, S, S1 and N peptides were pooled together: after overnight stimulation a reduced production of IFN-γ, was detected in FTY group compared to OCR and NAT groups (Kruskal-Wallis p<0,0001. Post-test analysis, OCR vs FTY p=0,0007 and FTY vs NAT p=0,0004 and OCR vs NAT p=ns) (**Figure 3C**). These results were confirmed after normalization of SARS-CoV-2 Pool peptide-induced to PHA-induced IFN-γ production (**Supplementary Data 1.1**)

Overall, a reduced T-cell IFN-γ response in PwMS treated with fingolimod was observed compared to patients treated with ocrelizumab or natalizumab (**Figure 3**).

Intracellular staining was performed on a subgroup of PwMS and HD to assess CD4+ and CD8+ T-cell specific production of IFN-γ upon SARS-CoV-2 peptide libraries stimulation with flow cytometry. The analysis showed that both CD4+ and CD8+ T-cells contributed to IFN-γ production in PwMS and HD (**Supplementary Data 1.2** and **Supplementary Figure 2.1**)

### Correlations

Anti-S antibody titers, IFN-γ production upon T-cell stimulation with S1, S and pooled peptides were correlated among them and with CD3+, CD4+, CD8+, CD19+ and CD16+CD56+ absolute counts as well as CD4/CD8 ratio, in the global sample and in each subgroup of PwMS.

**Figure 4:**
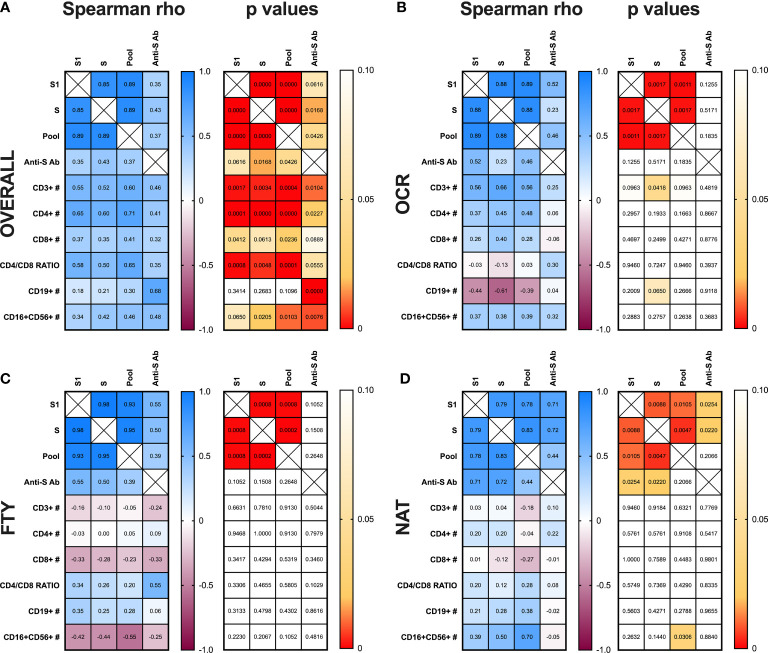
Correlation analysis between T-, B-cell responses and peripheral blood lymphocyte subset absolute counts. Spearman’s correlation analysis between IFN-γ production upon S1, S, and pooled peptide (Pool) stimulation of whole blood, anti-S antibody (Ab) titers, T- (CD3+, CD4+, CD8+), B- (CD19+), NK- (CD16+CD56+) cell absolute counts and CD4/CD8 ratio in peripheral blood are represented for the overall cohort of PwMS **(A)** and the three subgroups of subjects on ocrelizumab **(B)**, fingolimod **(C)** and natalizumab **(D)** treatment. **(A–D)**. Spearman rho coefficient are reported in the left grid while the corresponding p values are represented in the right grid for each panel of the figure. PwMS, people with multiple sclerosis; OCR, ocrelizumab group; FYT, fingolimod group; NAT, natalizumab group.

The amount of IFN-γ production after stimulation with S1, S and pooled peptide libraries were reciprocally correlated, (Spearman r: >0,8 and p<0,0001) (**Figure 4A**). Anti-S antibody titers were positively correlated with IFN-γ production upon S and pooled peptide stimulation (Spearman r: 0,43 and 0,37; p=0,0168 and 0,0426, respectively) (**Figure 4A**). Furthermore, CD3+, CD4+ and CD8+ absolute counts and CD4/CD8 ratio were strongly and directly correlated with IFN-γ production upon Spike peptides (both S and S1) stimulation, while anti-S antibody production was positively correlated with CD19+ cell absolute counts. Finally, CD16+CD56+ lymphocyte absolute counts were directly correlated to both IFN-γ production upon S and pooled peptide stimulation, and with anti-S antibody titers (**Figure 4A**).

Proceeding with the correlation analysis in each subgroup of PwMS, in the OCR group, IFN-γ production upon S stimulation was directly correlated with CD3+ absolute counts (Spearman r: 0,66; p=0,0418). Moreover, IFN-γ production upon stimulation with S1 and pooled peptides tended to be directly correlated with CD3+ absolute counts (Spearman r: 0,56; p=0,0963 for both) (**Figure 4B**).

In the FTY group no correlations were found between anti-S antibody titers, IFN-γ production after stimulation with S1, S and pooled peptide libraries and peripheral blood lymphocyte absolute counts (**Figure 4C**).

In the NAT group, anti-S antibody titers were directly correlated to IFN-γ production after S1 and S peptide stimulation (Spearman r: 0,71; p= 0,0254 and Spearman r: 0,72; p= 0,0220, respectively). Moreover, IFN-γ production after pooled peptide stimulation was directly associated to CD16+CD56+ lymphocyte absolute counts (Spearman r: 0,70; p= 0,0306) (**Figure 4D**).

## Discussion

In the ongoing SARS-CoV-2 pandemic scenario, the quick development of specific vaccines raised the need to better characterize the efficacy and immunological responses to vaccines in fragile subjects, such as PwMS receiving DMTs. Up today, vaccine responses have been evaluated mainly through the analysis of anti-Spike or anti receptor binding domain (RBD) antibody production and the evaluation of the SARS-CoV-2 neutralizing sera titers. Very little is known about cell-mediated responses, CD8+ cytotoxic activity and their potential protective role. In PwMS, it has been already shown that subjects receiving ocrelizumab and fingolimod develop reduced or absent anti SARS-CoV-2 specific antibody titers after either vaccination or natural infection (8, 9, 13). Therefore, we evaluated T-cell response to SARS-CoV-2 vaccine in PwMS receiving ocrelizumab, fingolimod and natalizumab after two-dose vaccination with Comirnaty^®^. Our results clearly demonstrated different patterns of immunological responses in the three group of patients.

As expected, PwMS receiving ocrelizumab, had reduced B-cell absolute counts and impaired anti-S antibody titers. Conversely, after T-cell stimulation, IFN-γ levels in supernatants were comparable to those observed in PwMS on natalizumab and in HDs. The VELOCE study has already demonstrated an attenuated humoral response to clinical relevant vaccines and neoantigens in subjects on ocrelizumab treatment (16). Recently, Apostolidis et al. demonstrated that although antibody responses to SARS-CoV-2 were impaired in most of PwMS treated with ocrelizumab after mRNA vaccine administration, T-cell priming, especially of T helper-1 and CD8 T-cells was completely intact (17). Moreover, Brill et al. showed that PwMS on ocrelizumab generated comparable SARS-CoV-2–specific T-cell responses with healthy controls, despite lower antibody response following vaccination (18). Our data confirm these previous observations, showing the preserved ability of T-cell to produce IFN-γ after stimulation with SARS-CoV-2 specific peptides, despite the impairment of B-cell absolute counts and decreased anti-S antibody production induced by ocrelizumab.

A different pattern of immunological response was observed in subjects receiving fingolimod, in whom both humoral and cell-mediated responses were impaired. Previous studies showed that although most fingolimod-treated PwMS could mount humoral immune responses against novel and recall antigens, response rates were reduced compared to placebo-treated patients (19). Moreover, Mehling et al. showed that after seasonal influenza vaccination, PwMS treated with fingolimod were able to mount humoral and cell-mediated immune responses, which were comparable to healthy controls (20). In contrast with these observations, we have found reduced T-cell responses to SARS-CoV-2 mRNA vaccines in PwMS on fingolimod. These findings might be explained, considering the mechanisms of action of this modulator of the sphingosine-1-phosphate receptor, that blocks the egress of lymphocytes from secondary lymphoid organs, and reduces peripheral blood lymphocyte count, in a dose dependent manner. We can speculate that the impaired T-cell response observed in the study population was due to the absence of primed T-cells in peripheral blood, which were retained in the lymph nodes. According to some studies, low-dose fingolimod can act as a facilitator to establish primary T-cell responses, increasing the probability of naïve T-cells to encounter antigen presenting cells in the lymph nodes and be primed and activated (21). Therefore, fingolimod could reduce circulating SARS-CoV-2 specific T-cell in peripheral blood, while their number could be unchanged or even increased inside lymph nodes. Clinical implications of this hypothesis should be verified in longitudinal cohort of vaccinated PwMS receiving fingolimod treatment, trying to assess the rate of infections and disease severity after SARS-CoV-2 vaccination. Indeed, observational studies have shown that treatment with fingolimod is not a risk factor for severe COVID-19 in PwMS infected by SARS-CoV-2 (22–24).

Another different pattern of immunological response to SARS-CoV-2 mRNA vaccines was observed in PwMS treated with natalizumab. These subjects showed normal B- and T-cell responses, comparable to those observed in healthy donors. Our results confirm previous observation showing an efficient humoral immune response to influenza, tetanus toxoid and mRNA COVID-19 vaccines in PwMS on natalizumab (25–27). Little is known about T-cell response after vaccine administration in these subjects. However, considering the effect of natalizumab in interfering with the migration of T-lymphocyte expressing the alpha-4 integrin from the peripheral blood into the central nervous system, no concerns have been raised on the ability to mount a peripheral T-cell mediated immunity, as confirmed by our data.

The presence of robust SARS-CoV-2-specific CD4+ and CD8+ T-cell responses has been associated with lower COVID-19 severity, and seems to contribute in resolving acute disease (28). Furthermore, McMahan et al. demonstrated that the depletion of CD8+ T-cells in convalescent rhesus macaques, partially abrogated the protective efficacy of natural immunity against rechallenge with SARS-CoV-2 (12). Moreover, Bange et al. demonstrated that in patients with hematologic malignancies with reduced SARS-CoV-2 -specific antibody responses and increased COVID-19 associated mortality, a greater number of CD8+ T-lymphocytes was associated to a better outcome. The authors showed also that 77% of hematologic patients had detectable SARS-CoV-2-specific T-cell responses. Taken together these results suggest a protective role of specific CD8+ T-cells in patients with hematologic malignancy who recovered from SARS-CoV-2 infection (29).

One limitation of this study is its cross-sectional design, considering the dynamic changes of SARS-CoV-2 immune responses over time after vaccination. It has been demonstrated that circulating antibodies induced by mRNA vaccine as well as specific T-cell responses (such as IFN-γ and IL-2 production) persist at least 6 months post-vaccination, though there is some decay over time. Indeed, both anti-S titers and CD4+ and CD8+ T-cell activation and cytokine production usually peak 1 week after mRNA vaccine recall dose and tend to slightly decrease after 3-6 months post second dose administration (30–32). Furthermore, Shine et al. demonstrated stable levels of CD4+ and CD8+ IFN-γ and IL-2 production upon S-pool peptide stimulation from 40 to 85 days post vaccination (31). A robust spike-specific CD8+ T-cell response was detected early after the first dose of mRNABNT162b2, which was stable after the second dose, and still detectable after 3-4 months after completing the vaccination schedule. Therefore, although in our study anti-S antibody titers and specific T-cell responses were assessed after a variable period after mRNABNT162b2 vaccine second dose, ranging from 34 to 106 days, with a median of 73 days, this period corresponds to the phase in which B- and T-cell responses seem to be stable or slowly decreasing. Moreover, anti-S antibody titers and specific T-cell responses were assessed after a median of 46 [IQR=39-73], 73 [IQR=60-82] and 83 [IQR=64-96] days from mRNABNT162b2 second dose in OCR, FTY and NAT groups, respectively. Therefore, in PwMS on natalizumab (for whom a normal immunological response was expected) sampling time was slightly longer compared to PwMS on fingolimod and ocrelizumab. However, PwMS on natalizumab had higher anti-S antibody titers compared to PwMS on ocrelizumab and higher anti-S antibody titers and specific T-cell responses compared to PwMS on fingolimod. This evidence supports the hypothesis of an impaired immunological response in PwMS on ocrelizumab (limited to anti-S antibody production) and fingolimod (involving both anti-S antibody production and specific T-cell activation), compared to PwMS on natalizumab. A further confirm of this hypothesis was obtained after resampling part of the cohort of PwMS and HD to perform T-cell stimulation and assess anti-S antibody titers with a longitudinal approach. No statistically significant differences were found after comparing anti-S antibody titers and IFN-γ production upon S1, S and Pool stimulation of peripheral blood T-cells at the two different timepoints, 114 days apart, probably because of the limited number of subjects included in the analysis (**Supplementary Data 1.3** and **Supplementary Figure 2.2**).

Our observations suggest that SARS-CoV-2-specific CD4+ and CD8+ T-cell responses should be considered to better evaluate vaccination and public health strategies, and assessed together with neutralizing antibody titers to predict protection from infection and severe COVID-19.

In this scenario, here we present an affordable and accessible approach to evaluate cell-mediated immune response using a SARS-CoV-2 IGRA test, based on whole blood stimulation with specific peptides libraries. The same method was also adopted by other groups in different settings (33).

All the patients included in this study tested negative for the detection of anti-N antibodies and did not report any prior symptom of COVID-19 disease. However, some patients showed IFN-γ production upon N peptide stimulation even if at low levels. Only two patients showed IFN-γ production comparable with levels detected in ocrelizumab treated MS subjects healed from COVID-19, previously studied by our group (12). This could be due to a previous SARS-CoV-2 infection, which cannot be completely rule out in PwMS on ocrelizumab and fingolimod, even if anti-N antibody tested negative, considering the impairment of specific antibody production in these patients, or to cross-reactivity with other coronaviruses (34). Although this can represent a limitation of the IGRA test, we believe that the results of the study were not influenced by this phenomenon, assuming an equal rate of cross-reactive responses in the three groups of patients.

The correlation analysis in the global cohort of PwMS showed a strong positive correlation between peripheral blood T-lymphocyte absolute counts (especially total CD3+ and CD4+ and CD4/CD8 ratio) and IFN-γ production after Spike peptide stimulation as well as a strong positive association between peripheral blood B-lymphocyte absolute counts and anti-S titers. Therefore, the evaluation of peripheral blood B- and T-lymphocyte counts can help in predicting the humoral and cell-mediated responses to mRNA vaccines in PwMS on DMTs.

The study has some limitations, namely the small sample size, the cross-sectional approach and the use of a mRNA vaccine only, which reduces the possibility to extend our considerations to other settings, such as subject who received adenovirus vector-based vaccines for SARS-CoV-2.

Larger cohorts of PwMS receiving different DMTs and vaccinated with different methods are needed to confirm our results. Furthermore, longitudinal studies should be designed to assess the duration of both humoral and cell mediated immunity in specific populations of immunosuppressed subjects, such as PwMS on DMTs. Longitudinal studies on larger cohorts will also allow to correlate immune responses with protection from SARS-CoV-2 infection and severe COVID-19.

## Data Availability Statement

The original contributions presented in the study are included in the article/**Supplementary Material**. Further inquiries can be directed to the corresponding author.

## Ethics Statement

The studies involving human participants were reviewed and approved by Comitato Etico Indipendente Fondazione PTV Policlinico Tor Vergata. The patients/participants provided their written informed consent to participate in this study.

## Author Contributions

MI: Drafting/revision of the manuscript for content, including medical writing for content, major role in the acquisition of data, study design, and analysis and interpretation of data. DL: Revision of the manuscript for content, including medical writing for content, and major role in the acquisition of data. GC: Major role in the acquisition of data. LaCa: Drafting/revision of the manuscript for content, including medical writing for content, and analysis and interpretation of data. VM: Major role in the acquisition of data, and study design. ET: Study design and analysis and interpretation of data. LuCo: Major role in the acquisition of data, and analysis and interpretation of data. AL: Major role in the acquisition of data, and analysis and interpretation of data. DF: Major role in the acquisition of data, and study design. FB: Major role in the acquisition of data, and study design. SG: Major role in the acquisition of data. MM: Major role in the acquisition of data. MAZ: Major role in the acquisition, analysis and interpretation of data. MRC: Major role in the acquisition, analysis and interpretation of data. RN: Revision of the manuscript for content, including medical writing for content. SB: Revision of the manuscript for content, including medical writing for content. MA: Revision of the manuscript for content, including medical writing for content, and analysis and interpretation of data. GM: Revision of the manuscript for content, including medical writing for content, and analysis and interpretation of data. LS: Drafting/revision of the manuscript for content, including medical writing for content, study concept or design, and analysis or interpretation of data. All authors contributed to the article and approved the submitted version.

## Funding

This study was supported by the NATO FRAMEWORK PROGRAMME SPS PROJET #G5817.

## Conflict of Interest

MI received honoraria for lectures from Biogen Italia, AIM Educational, MICOM srl. DL received travel funding from Biogen, Merk-Serono, Sanofi-Genzyme, Teva, speaking or consultation fees from Sanofi-Genzyme, Merk-Serono, Teva, Biogen, Novartis, Roche. LC received honoraria for lectures from MICOM srl. VM received honoraria for lectures from Janssen-Cilag. ET received honoraria for lectures from Gilead, AbbVie and MSD, and research grants from Gilead, outside the submitted work. FB received honoraria for lectures from Novartis. MA reports honoraria for lectures and research grants from Merk, Gilead, Abbvie, Angelini SpA, outside the submitted work. GM is an Advisory Board member of Biogen Idec, Genzyme, Merck-Serono, Novartis, Teva and received honoraria for speaking or consultation fees from Almirall, Bayer Schering, Biogen Idec, Merck Serono, Novartis, Sanofi-Genzyme, Roche, Mylan, Teva. LS reports honoraria for lectures and research grants from Merk, Gilead, Abbvie, Angelini SpA, outside the submitted work.

The remaining authors declare that the research was conducted in the absence of any commercial or financial relationships that could be construed as a potential conflict of interest.

## Publisher’s Note

All claims expressed in this article are solely those of the authors and do not necessarily represent those of their affiliated organizations, or those of the publisher, the editors and the reviewers. Any product that may be evaluated in this article, or claim that may be made by its manufacturer, is not guaranteed or endorsed by the publisher.
