# Supplementary material for: B- and T-Cell Responses After SARS-CoV-2 Vaccination in Patients With Multiple Sclerosis Receiving Disease Modifying Therapies: Immunological Patterns and Clinical Implications
Source: Front Immunol. 2022 Jan 17;12:796482. doi: 10.3389/fimmu.2021.796482 (PMC8801814; doi:10.3389/fimmu.2021.796482)
Supplement: Supplementary file 1 [file DataSheet_1.docx]

Supplementary Material

# Supplementary Data

- 1. **Normalization of SARS-CoV-2 specific peptide induced to PHA-induced IFN-γ production in PwMS and HD**

IFN-γ production upon SARS-CoV-2 specific stimulation (S, S1 and Pool) was normalized to the corresponding IFN-γ production upon PHA stimulation. The theoretical basis of this approach is that SARS-CoV-2 peptide to PHA ratio can eliminate the impact of individual immune variation on the IGRA test. Patients with immunocompromised conditions (such as PwMS on DMTs) have decreased IFN-γ production upon both SARS-CoV-2 peptide stimulation as well as PHA stimulation. This method has already been proposed for IGRA assay to improve the differential diagnosis of latent and active tuberculosis (15). Considering S1/PHA ratio, the values observed in FTY group were reduced compared to OCR and NAT groups (Kruskal-Wallis p=0,003. Post-test analysis, OCR vs FTY p=0,0027 and FTY vs NAT p=0,064 and OCR vs NAT p=ns). Concerning S/PHA ratio, the values observed in FTY group were reduced compared to NAT group (Kruskal-Wallis p=0,0431. Post-test analysis, OCR vs FTY p=ns, FTY vs NAT p=0,05 and OCR vs NAT p=ns). Considering Pool/PHA ratio, the values observed in FTY group were reduced compared to OCR and NAT groups (Kruskal-Wallis p=0,0005. Post-test analysis, OCR vs FTY p=0,002 and FTY vs NAT p=0,0025 and OCR vs NAT p=ns).

- 1. **Intracellular staining for the detection of IFN-γ production by CD4+ and CD8+ T-cells after SARS-CoV-2 peptide stimulation**

Intracellular staining for the detection of IFN-γ production by stimulated CD4+ and CD8+ T-cells from 4 PwMS (2 on natalizumab and 2 on fingolimod) and 4 HD was performed. Fresh heparinized whole blood was used for immunomagnetic separation of CD45+ leukocytes, using the StraightFrom® Whole Blood CD45 MicroBeads and Whole Blood Columns, all purchased from Miltenyi Biotec, following manufacturer instructions. Separated peripheral blood CD45+ leukocytes were washed in phosphate-buffered saline (PBS) solution and resuspended in RPMI medium supplemented with 10% of fetal bovine serum (FBS) at 10^7^ cells/ml. For each subject, 200µl of the cellular suspension were stimulated with the PepTivator SARS-CoV-2 Prot_S1 (S1), Prot_S (S) and pooled peptide (Pool) (consisting of PepTivator SARS-CoV-2 Prot_S1 + Prot_S + Prot_N) at a final concentration of 1µg/ml, in a 96-well round-bottom plate. A non-stimulated (NS), and PHA stimulated condition (5µg/ml) was also included for each subject. Brefeldin-A was added after the first 2-hours of incubation at a final concentration of 5µg/ml. After overnight incubation at 37°C, 5% CO2, 80% humidity, cells were harvested and washed in PBS solution supplemented with 1% FBS (stain buffer) and stained with the Zombie Aqua fluorescent viability dye (Biolegend) for 20 minutes at room temperature. Surface staining with anti-CD3 PerCp (clone SK7), anti-CD4 APC-Cy7 (clone RPA-T4) and anti-CD8 PE-Cy7 (clone SK1) was performed for 20 minutes at 4°C. Cells were washed twice with stain buffer and fixed for 30 minutes at room temperature and then permeabilized using the Cytofix/Cytoperm buffer (BD Bioscences). Intracellular staining was performed using anti-IFN-γ Pacific Blue (clone 4S.B3) monoclonal antibody, for 45 minutes at 4°C and then cells were washed twice with the permeabilization buffer and resuspended in PBS supplemented with 1% paraformaldehyde. All the monoclonal antibodies were purchased from Biolegend. Samples were acquired on a MACSQuant Analyzer 10 Flow Cytometer (Miltenyi Biotec). Data were analyzed using FlowJo v10 (BD Bioscences). Gating strategy is shown in Supplementary figure 2.1A. The percentages of cells producing IFN-γ in the NS condition was subtracted to SARS-CoV-2 peptide stimulated and PHA stimulated conditions (Supplementary figure 2.1B).

The analysis of the data showed that in PwMS and HD both CD4+ and CD8+ T-cells contributed to IFN-γ production upon S, S1 and Pool stimulation. PwMS on fingolimod showed lower percentages of IFN-γ producing CD4+ and CD8+ T-lymphocytes upon SARS-CoV-2 specific peptide stimulation, compared to PwMS on natalizumab (for CD4+IFN-γ+ median values, S1: 0,04% vs 0,49% vs 0,22%; S: 0,25% vs 0,55% vs 0,28%; Pool: 0,17% vs 0,85% vs 0,18%, for HD, NAT and FTY, respectively. For CD8+IFN-γ+, S1: 0,02% vs 0,47% vs 0,19%; S: 0,01% vs 0,44% vs 0,28%; Pool: 0,10% vs 0,64% vs 0,11%, for HD, NAT and FTY, respectively). Conversely, IFN-γ producing CD4+ and CD8+ T-lymphocytes seem to be preserved after PHA stimulation in PwMS on fingolimod compared to PwMS on natalizumab and HD (for CD4+IFN-γ+ median values, PHA: 0,21% vs 0,18% vs 0,64%; for CD8+IFN-γ+, PHA: 0,24% vs 0,21% vs 0,27%; for HD, NAT and FTY, respectively).

# Longitudinal evaluation of Anti-Spike antibody titers and specific T-cell responses in people with multiple sclerosis and healthy donors

Eight PwMS (F/M=7/1, 4 on fingolimod and 4 on natalizumab) and 4 HD (F/M=3/1) were resampled after a median [interquartile range (IQR)] of 193 [189-194] days, 187 [177-197] days and 282 [280-282] days after the mRNABNT162b2 vaccine second dose, respectively. We could not resample patients on ocrelizumab before the administration of the booster (third) dose of the mRNABNT162b2 vaccine. After resampling, T-cell stimulation with Spike (S) and nucleoprotein (N) peptide libraries (PepTivator SARS-CoV-2 Prot_S1, Prot_S and Prot_N) and Anti-S titers were assessed. Concerning T-cell specific responses, IFN-γ production was reduced in PwMS on fingolimod compared to PwMS on natalizumab upon S1, S and pooled (Pool) peptide stimulation (Mann-Whitney p=0,029, 0,057 and 0,057, respectively). Furthermore, IFN-γ production was also reduced in PwMS on fingolimod compared to HD upon S1, S and Pool stimulation (Mann-Whitney p=0,029, 0,029 and 0,029, respectively). No differences were observed when comparing PHA stimulation in the three groups (Supplementary figure 1A-D). Considering the variability observed in PHA stimulation over time in the same subject, IFN-γ production after SARS-CoV-2 specific stimulation (S, S1 and Pool) were “normalized” to the levels of IFN-γ produced upon PHA stimulation. In this case also, the ratios for IFN-γ production (S1/PHA, S/PHA and Pool/PHA) observed in PwMS on fingolimod were reduced compared to PwMS on natalizumab (Mann-Whitney p= 0,029, 0,057 and 0,057, respectively) and HD (Mann-Whitney p= 0,086, 0,057 and 0,057, respectively) (Supplementary figure 1E-G).

Concerning specific Anti-S antibodies at resampling, PwMS on fingolimod had reduced titers compared to PwMS on natalizumab (Mann-Whitney p=0.029) and HD (Mann-Whitney p=0.029) (Supplementary figure 1H).

For each subgroup, IFN-γ levels detected upon S1, S and Poll stimulation at T1 were compared to the corresponding levels measured at T2 (Wilcoxon matched-pairs signed rank test for non-parametric data). No statistically significant differences were observed in FTY, NAT groups and in HD (Supplementary figure1A-C). Moreover, no statistically significant differences were found after normalizing IFN-γ production upon SARS-CoV-2 specific stimulation (S, S1 and Pool) to the levels of IFN-γ production upon PHA stimulation (Supplementary figure 1E-G). Anti-S antibody levels measured at T1 and T2 were compared and although there was a general reduction of anti-S titers in PwMS and HD, the difference did not reach the statistically significance, probably because of the limited number of subjects resampled at T2 (Supplementary figure 1H).

1. **Supplementary Figures and Tables**
   1. **Supplementary Figure: Intracellular staining for the detection of IFN-γ production by CD4+ and CD8+ T-cells after SARS-CoV-2 peptide stimulation**

**
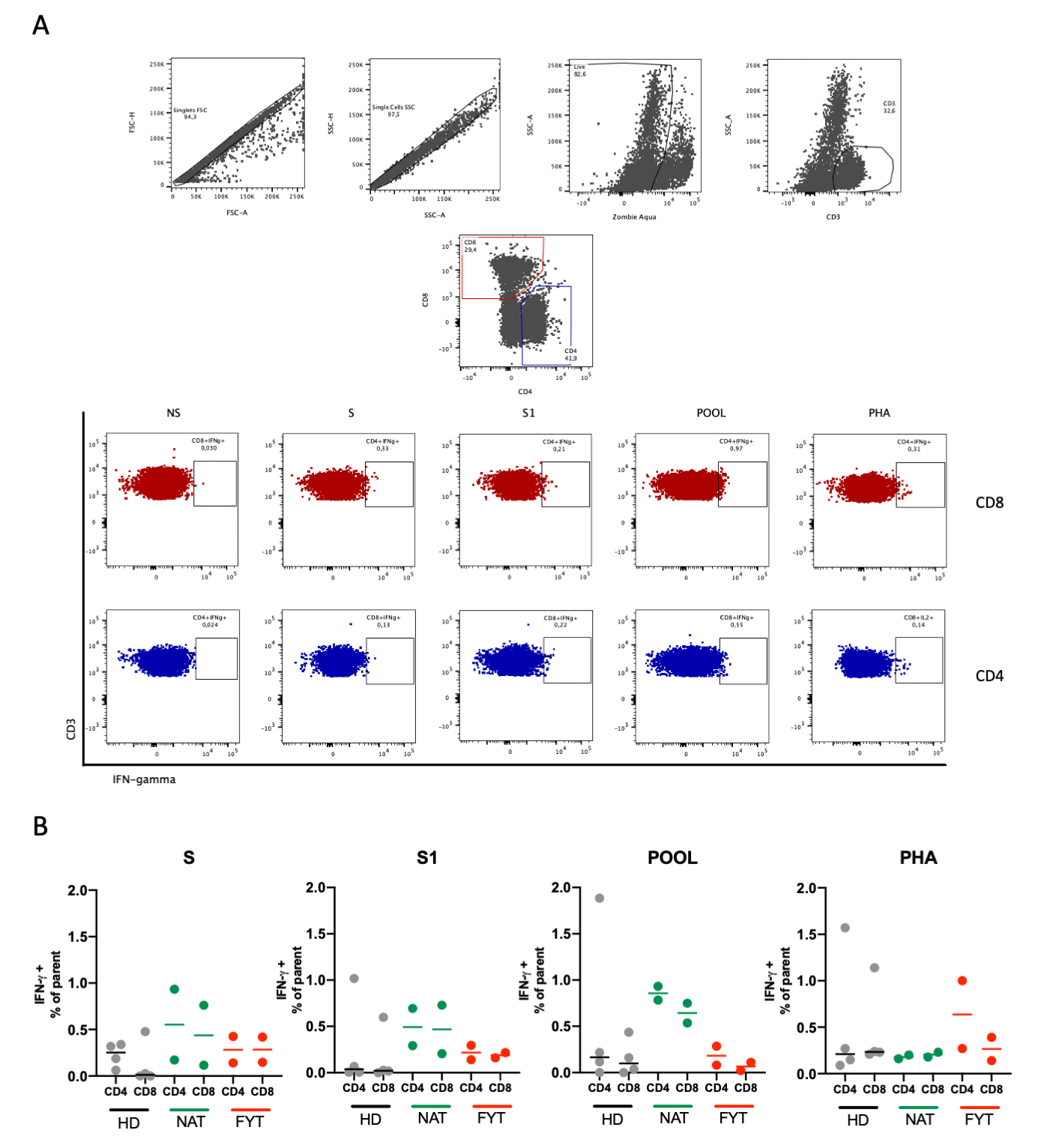
**

(A) Gating strategy for intracellular staining of IFN-γ production upon SARS-CoV-2 peptide stimulation by CD4+ and CD8+ T-cells. After doublets exclusion using the FSC-A and -H and SSC-A and -H parameters, viable cells were gated, excluding death cells through the Zombie Aqua fixable viability dye. After gating CD3+ lymphocytes, CD3+CD4+ and CD3+CD8+ cells were identified. For each T-lymphocyte subpopulation IFN-γ production was analyzed and expressed as the percentages of IFN-γ positive events of the parental population.

(B) For each subject IFN-γ production by CD4+ and CD8+ T-cells in non-stimulated (NS) PepTivator SARS-CoV-2 Prot_S1 (S1), Prot_S (S), pooled peptide (Pool) (consisting of Prot_S1 + Prot_S + Prot_N) and phytohemagglutinin (PHA) conditions were represented. The percentages of IFN-γ producing cells in the NS condition was subtracted to SARS-CoV-2 peptide- and PHA-stimulated conditions. HD are represented in gray, NAT in green and FTY in red. Horizontal bars represent median values.

- 1. **Supplementary Figure 1: Longitudinal evaluation of T-cell responses to SARS-CoV-2 Spike peptide libraries and anti-S antibody titers**


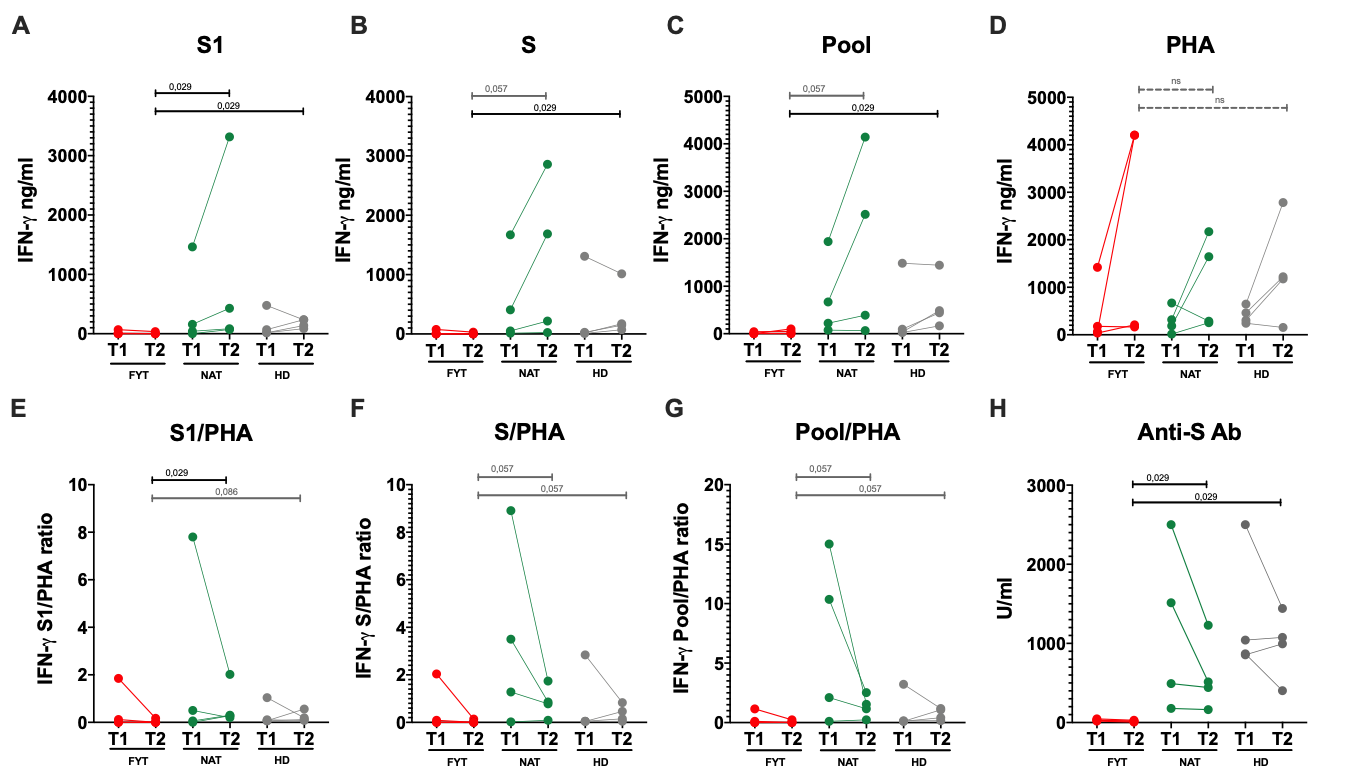


(A-D) IFN-γ production upon S1, S, pooled peptide (Pool=S1+S+N) and phytohemagglutinin (PHA) is represented for PwMS on fingolimod (FYT) in red, PwMS on natalizumab (NAT) in green and healthy donors (HD) in gray.

(E-G) IFN-γ production after SARS-CoV-2 specific stimulation (S, S1 and Pool) was “normalized” to the levels of IFN-γ production upon PHA stimulation, and ratios were represented for PwMS on fingolimod (FYT) in red, PwMS on natalizumab (NAT) in green and healthy donors (HD) in gray.

(H) Anti-S antibody titers are represented for PwMS on fingolimod (FYT) in red, PwMS on natalizumab (NAT) in green and healthy donors (HD) in gray.

For each subject two timepoints (T1 and T2), 114 [IQR:114-116] days apart, are represented. The differences between FYT vs NAT, FYT vs HD and NAT vs HD groups at T2 were assessed using the Mann-Whitney test for non-parametric quantitative data.
